# PhenoMiner: a quantitative phenotype database for the laboratory rat, *Rattus norvegicus*. Application in hypertension and renal disease

**DOI:** 10.1093/database/bau128

**Published:** 2015-01-28

**Authors:** Shur-Jen Wang, Stanley J. F. Laulederkind, G. Thomas Hayman, Victoria Petri, Weisong Liu, Jennifer R. Smith, Rajni Nigam, Melinda R. Dwinell, Mary Shimoyama

**Affiliations:** ^1^Human and Molecular Genetics Center, Medical College of Wisconsin, ^2^Department of Physiology, Medical College of Wisconsin and ^3^Department of Surgery, Medical College of Wisconsin, 8701 Watertown Plank Road, Milwaukee, WI53226, USA

## Abstract

Rats have been used extensively as animal models to study physiological and pathological processes involved in human diseases. Numerous rat strains have been selectively bred for certain biological traits related to specific medical interests. Recently, the Rat Genome Database (http://rgd.mcw.edu) has initiated the PhenoMiner project to integrate quantitative phenotype data from the PhysGen Program for Genomic Applications and the National BioResource Project in Japan as well as manual annotations from biomedical literature. PhenoMiner, the search engine for these integrated phenotype data, facilitates mining of data sets across studies by searching the database with a combination of terms from four different ontologies/vocabularies (Rat Strain Ontology, Clinical Measurement Ontology, Measurement Method Ontology and Experimental Condition Ontology). In this study, salt-induced hypertension was used as a model to retrieve blood pressure records of Brown Norway, Fawn-Hooded Hypertensive (FHH) and Dahl salt-sensitive (SS) rat strains. The records from these three strains served as a basis for comparing records from consomic/congenic/mutant offspring derived from them. We examined the cardiovascular and renal phenotypes of consomics derived from FHH and SS, and of SS congenics and mutants. The availability of quantitative records across laboratories in one database, such as these provided by PhenoMiner, can empower researchers to make the best use of publicly available data.

**Database URL:** http://rgd.mcw.edu

## Introduction

Rats have long been one of the preferred animal models for the study of human diseases. Many rat strains have been selectively bred for resistance/susceptibility to various diseases such as cardiovascular diseases, renal diseases and obesity/metabolic syndrome. For example, Dahl salt- sensitive (SS) inbred rats were selectively bred from Sprague-Dawley outbred rats for sensitivity to salt-induced hypertension. After developing hypertension, the SS rats progress to proteinuria and kidney pathologies (1,2). The Fawn-Hooded Hypertensive (FHH) rat is another genetic model for hypertension and renal diseases. The development of hypertension and renal disease progresses slowly over the lifetime of FHH rats (3) and high-salt-fed FHH rats exhibit a greater sensitivity to hypertension and renal disease (4). A common approach to examine genetic mechanisms controlling hypertension susceptibility is to cross susceptible and resistant strains and characterize the genomic composition of resistant offspring. Brown Norway (BN) and Lewis (LEW) are two common resistant strains that have been used as donors to rescue the hypertensive phenotype of susceptible rat strains (5). The PhysGen Program for Genomic Application (http://pga.mcw.edu) and the PhysGen Knockout project (http://www.rgd.mcw.edu/wg/physgenknockouts) have produced large-scale phenotype data from breeding resistant and susceptible rats. These quantitative phenotype data of wild-type, consomic and mutant rats are available online through PhysGen (http://pga.mcw.edu/). Data in PhysGen are categorized by study projects (Consomic or ENU mutant) with limited searching flexibility. To better facilitate data mining across multiple projects, the Rat Genome Database (RGD) has initiated the PhenoMiner project to integrate quantitative phenotype data from PhysGen projects (http://pga.mcw.edu/) and the National BioResource Project in Japan (NBRP) (http://www.anim.med.kyoto-u.ac.jp/nbr/) (6). The high-throughput data from PhysGen and NBRP are represented by more than 60 000 records in the PhenoMiner user interface (7). In addition to the high-throughput imported data, there are manually curated records in PhenoMiner. These annotations are manually curated from literature previously curated for rat strains and quantitative trait loci at RGD. The process of literature curation has covered cardiovascular phenotypes, cancer, diabetes, renal function and inflammatory processes (8).

The curated quantitative data in PhenoMiner are systematically organized by four ontologies: rat strain ontology (RS) (9), clinical measurement ontology (CMO), measurement method ontology (MMO) and experimental condition ontology (XCO) (10,11). Each CMO record is defined by how the record was measured (MMO), under what XCO and in which RS. The RS ontology delineates relationships among rats derived from the same ancestors. For example, the standard nomenclature identifies SS/Jr (RGD: 10041) as a child strain of the parent SS (RGD: 69369) and as the parent from which two sibling strains SS/JrHsd (RGD: 1582190) and SS/JrMco (RGD: 724573) were derived. Following the RS Ontology, the kinship among rats used in different studies can be mapped out for the benefit of future study design.

Using salt-induced hypertension as a model, we examined the blood pressure and renal function records in BN, FHH, SS and their derivatives from selective breeding. This article with data retrieval and analysis demonstrates the searching functionality and analytical application of curated data in PhenoMiner.

## Methods

**Figure 1.**
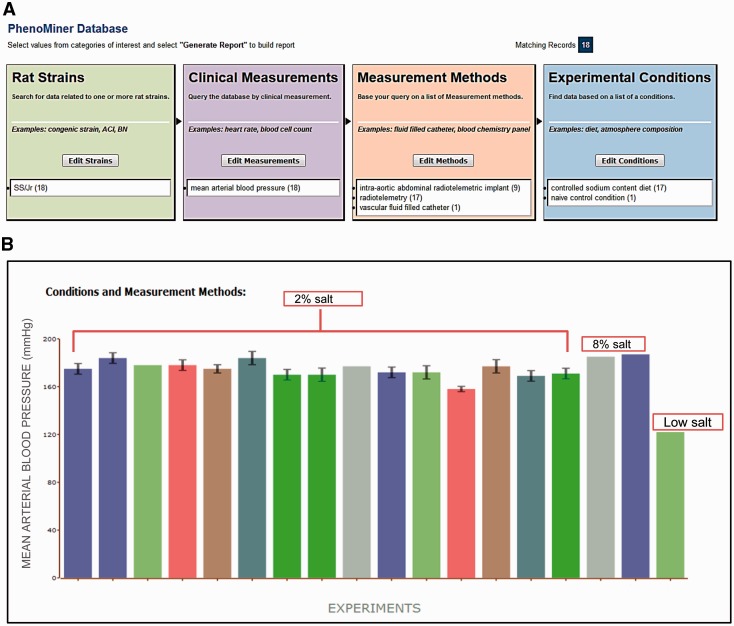
(**A**) Final search page before generating a report in PhenoMiner. A total of 18 records were retrieved by limiting data to four ontologies: Rat Strain, Clinical Measurement, Measurement Method and Experimental Condition. The record distribution in each ontology is shown in parentheses following the term. (**B**) Chart display of MAP data set generated in PhenoMiner. These records are from SS/Jr (RGD: 10041) on controlled sodium content diets: low, 2%, or 8%. Each bar represents one experiment and the experimental conditions and measurement methods associated with the experiment are color coded and explained under “Conditions and Measurement Methods.” To simplify the display, these experimental details of each bar are omitted. (Data accessed June 2014)

A video tutorial and step-by-step instructions for use of PhenoMiner can be found on the RGD website (http://www.rgd.mcw.edu/wg/home/rgd_rat_community_videos/introduction-to-the-rgd-phenotypes-and-models-portal) and in a recent publication (7). All data presented in this article can be downloaded from PhenoMiner (http://www.rgd.mcw.edu/phenotypes/) in the Phenotypes & Models Portal at RGD. Data in PhenoMiner are searchable across multiple studies by using the four ontologies: RS, CMO, MMO and XCO (Figure 1A). Users can select any ontology term to start the query. To retrieve the data shown in Figure 1, the SS/Jr inbred rat strain was first selected from the ‘inbred strain’ folder on the ‘Rat Strains Selection’ page (http://www.rgd.mcw.edu/phenotypes/termSelection/selectTerms?ont=RS). The data in Figure 1B compare only SS/Jr rats, not including the child strains, e.g. SS/JrHsd, SS/JrMco, SS/JrRkb or SS/JrSeac, so only records (∼410) directly associated with SS/Jr were selected. To mine out ‘mean arterial blood pressure’ records from these SS/Jr-associated records, ‘Limit By Clinical measurements’ was selected after the SS/Jr selection. In this example, a combination of two ontologies (RS and CMO) was sufficient to retrieve the desired records. The retrieved data are from multiple experiments and are displayed in a bar chart (Figure 1B) and tables for download. Users can customize the chart display by excluding certain ontologies/conditions or download the expanded data table for further analysis. More detailed information about vendor and feeding conditions are obtained from notes in the downloaded tables. The complete diet data can also be downloaded from PhysGen (http://pga.mcw.edu/). Data in PhysGen are grouped into ‘Consomic’ and ‘ENU Mutant’ projects. The mean arterial blood pressure (MAP) data can be downloaded from ‘Phenotype’ under ‘Consomic’ in the data section. The NBRP phenotype data for all strains are arranged into nine groups to allow users to search strain-associated data by groups. The data used in this article can also be found in the ‘Urine Volume and Urine Electrolyte Values’ group at NBRP Rat.

## Results

### Searches in PhenoMiner

To examine MAP records of SS strains, data were retrieved from PhenoMiner, RGD’s quantitative phenotype database (8). Data retrieval was started by searching PhenoMiner for the SS/Jr strain. By selecting one rat strain, one clinical measurement, three measurement methods and two experimental conditions (Figure 1A), 18 records curated from 14 publications were retrieved (Figure 1B). These MAP records were from rats maintained on low salt (naïve control), 2% or 8% controlled sodium content diets for various lengths of time. An expanded data table with all the record details, references and experimental notes is downloadable for further reference. Rat chow with high salt content (2% or 8%) raised MAP in SS/Jr when compared with low salt groups. However, the correlation between salt content and MAP levels was not linear among these data. The MAPs in SS/Jr rats on a 2% salt diet had a distribution from 169 to 184 mmHg, some of which were close to the MAPs in rats on an 8% salt diet (Figure 1B). Some of these variations could be resolved by examining experimental details such as animal age, measurement method or diet feeding scheme, from the expanded data table.

### Diet effects on MAP

**Figure 2.**
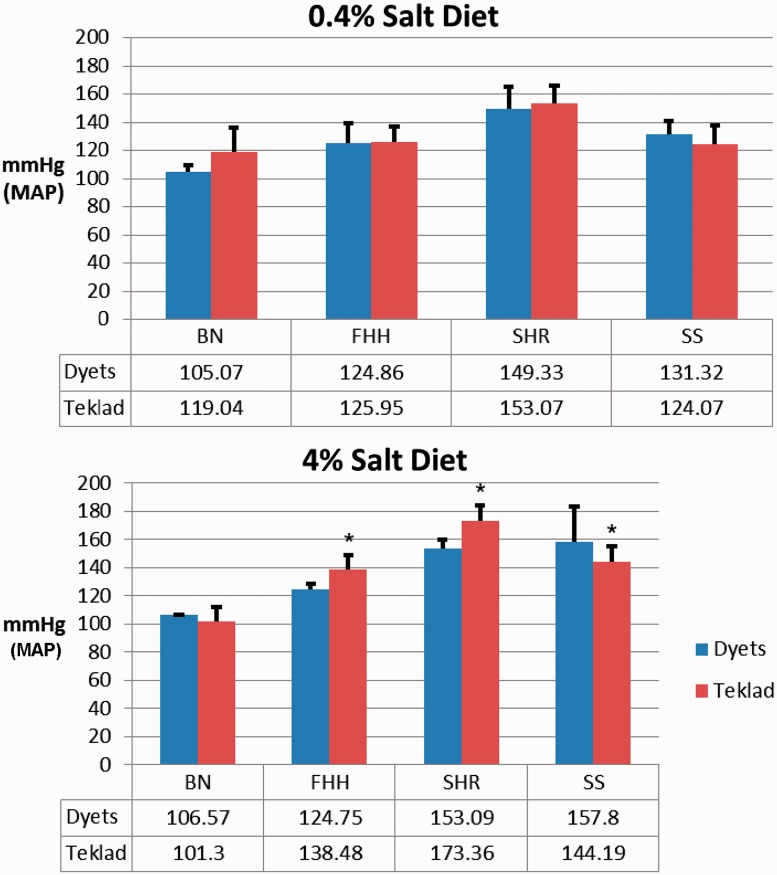
Effect of diet sources on MAP in four rat strains: BN, FHH, SHR and SS. Top: MAP was measured in rats on 0.4% salt-containing chows from Dyets or Teklad. Bottom: MAP was measured in rats on 4% salt-containing chows from Dyets or Teklad. Data for each strain were downloaded from PhenoMiner. The official nomenclature for the rat strains shown are BN/NHsdMcwi (BN), FHH/EurMcwi (FHH), SHR/NCrl (SHR) and SS/JrHsdMcwi (SS). Data tables underneath the charts show the mean values of each group. *P < 0.05 vs. same strain on Dyets chow.

Diet and genetic factors play roles in the development of hypertension. The hypertension phenotype of SS rats induced by high salt intake can be modified by diet (12). The PhysGen Program for Genomic Application (PhysGen) at the Medical College of Wisconsin carried out a study that carefully examined diet effects on hypertension and renal diseases (http://pga.mcw.edu). These data are now available through PhenoMiner. To retrieve MAP records of these PhysGen data, PhenoMiner searches started with selecting wild-type (parental) strains used in Physgen. The hypertension-resistant BN strain (BN/NHsdMcwi, RGD:61498) and three hypertension model strains, FHH (FHH/EurMcwi, RGD: 629509), spontaneously hypertensive rat (SHR/NCrlBR, RGD: 1357994) and SS (SS/JrHsdMcwi, RGD: 61499), were selected to be compared in Figure 2. Since the details of diet regimen were in the notes, the expanded data tables associated with searches were downloaded and filtered to create the bar charts shown in Figure 2. The MAPs were not significantly different among the four rat strains on 0.4% salt chow (Figure 2, top). However, with a high salt content (4%) chow, differential effects emerged, depending on the rat strains (Figure 2, bottom). The SS rats fed with 4% salt Dyets brand chow had higher MAPs than those fed with 4% salt Teklad chow. The effect of diet might explain some of the MAP variation among SS rats on 2% salt diets shown in Figure 1. The MAP variation caused by different rat chows is evident in PGA studies (http://pga.mcw.edu/?module=content&func=DataStatus).

### Hypertension renal disease model

High salt diets induce both hypertension and renal diseases in SS rats and FHH rats. To look at the effect of salt levels in diet on cardiovascular and renal phenotypes, related data of BN, FHH and SS were retrieved from PhenoMiner and organized into Table 1. With increased salt levels in the diet, from 4% to 8%, all three rat strains showed increased urine total protein excretion rate (UPE) and urine albumin excretion rate (UAE). SS rats, in addition to increased renal protein excretion, developed hypertension with increasing salt content in the diet. FHH rats developed hypertension and severe proteinuria (472.36 ± 209.46 mg/day) on an 8% salt diet plus L-NAME (NG-nitroarginine methyl ester). On the other hand, BN rats showed more resistance to hypertension and renal disease on the same diet (Table 1). Most of the NBRP phenotype data (Table 2) were collected under specific pathogen-free conditions, which are comparable to the low salt condition in Table 1. Table 2 lists the systolic blood pressure (SBP) measurements and urine measurements of two NBRP strains, BN/SsNSlc (RGD:1302631) and SHR/NCrlCrlj (RGD:629465). Although SBP was measured in NBRP rats instead of MAP, as measured in PhysGen rats, SHR rats exhibited higher blood pressure than the BN rats in both data sources. Sodium measurements were also different between these two sources. However, from ‘urine Na^+^ level’ and ‘urine volume’ in NBRP rats, we calculated the ‘Na^+^ excretion (mEq/day)’ for the two strains. The calculated Na+ excretion (mEq/day) for BN/SsNSlc can be referenced as a basal value to compare the Na^+^ measurements of BN rats under high salt conditions from PhysGen.

**Table 1.** MAP and urine measurements (UPE, UAE and Na^+^ excretion rate) in rats under different diet regimens

SS/JrHsdMcwi	Low salt	4% salt	8% salt	
MAP (mmHg)	131.32 ± 9.60	157.81 ± 25.64	176.81 ± 20.06	
UPE (mg/day)		132.70 ± 95.13	215.00 ± 116.41	
UAE (mg/day)		64.95 ± 62.02	82.65 ± 62.42	
Na^+^ excretion (mEq/day)		9.28 ± 5.33	14.23 ( 7.78	


BN/NHsdMcwi	Low salt	4% salt	8% salt	8% salt + L-NAME
MAP (mmHg)	105.07 ± 4.30	101.30 ± 6.14	110.83 ± 3.37	120.84 ± 7.37
UPE (mg/day)		10.61 ± 4.46	73.25 ± 37.22	32.25 ± 13.87
UAE (mg/day)		0.44 ± 0.22	4.05 ± 3.93	5.41 ± 2.85
Na+ excretion (mEq/day)		11.63 ± 3.17	25.20 ± 8.56	19.07 ± 4.62


FHH/EurMcwi	Low salt	4% salt	8% salt + L-NAME	
MAP (mmHg)	124.86 ± 14.50	124.75 ± 3.31	181.54 ± 20.73	
UPE (mg/day)		37.35 ± 16.90	472.36 ± 209.46	
UAE (mg/day)		12.24 ± 7.59	183.29 ± 96.23	
Na^+^ excretion (mEq/day)		13.82 ± 4.11	18.43 ± 5.95

**Table 2.** SBP, urine Na^+^ level and urine volume from two NBRP rat strains: BN/SsNSlc and SHR/NCrlCrlj

Clinical measurements	BN/SsNSlc	SHR/NCrlCrlj
SBP (mmHg)	137 ± 16	169 ± 18
Urine Na^+^ level (mEq/l)	155 ± 19.5	109 ± 22.2
Urine volume (ml/6 h)	4.6 ± 0.82	6.5 ± 1.27
Na^+^ excretion (mEq/day)^a^	2.852	2.834

^a^The Na^+^ excretion (mEq/day) values [= ‘urine Na^+^ level(mEq/L)’ × ‘urine volume (ml/6 h)’ × 4] of these two rat strains were calculated from ‘urine Na^+^ level’ and ‘urine volume’. These rats were kept in specific pathogen-free conditions with no diet manipulation. All the data are downloaded from the expanded data tables from PhenoMiner. Values are means ± standard deviation.

### Consomic rats

Blood pressure records of consomic rats were searched to find chromosome localizations of genes potentially involved in salt-induced hypertension and renal disease. Complete sets of BN chromosome-substituted consomics only exist among FHH and SS rats in PhenoMiner. In these studies, chromosomes from BN, a hypertension-resistant donor, were transferred to the FHH or SS background and their offspring were phenotyped under high salt diets for SS consomics or ‘high salt 8% + L-NAME’ for FHH consomics. Table 3 is a comparison of MAP, UPE and Na^+^ excretion in 14 consomic strains, 7 from the FHH background and 7 from the SS background, highlighted in publications (13,14). The transfer of chromosome 20 from BN to FHH attenuated the salt+L-NAME-induced hypertension and proteinuria. Transfer of chromosomes 1, 15 and 16, from BN to FHH decreased UPE to less than 50% of the parental value (Table 3). Among the SS consomics compared, substitution of BN chromosomes attenuated hypertension induced by both 4% and 8% salt diets, and substitution of chromosomes 1, 5 and 18 significantly improved proteinuria (Table 3). The complete data sets including other measurements of the compared consomics or data of the other consomics are available from PhenoMiner (http://www.rgd.mcw.edu/phenotypes/).

**Table 3.** MAP, UPE and Na^+^ excretion rate in consomic rat strains under different diet regimens

	Low salt, MAP (mmHg)	8% salt + L-NAME					
		MAP (mmHg)	UPE (mg/day)	Na+ excretion (mEq/day)			
FHH^a^	124.86 ± 14.50	181.54 ± 20.73	472.36 ± 209.46	18.43 ± 5.95			
FHH-1BN	126.2 ± 13.94	169.93 ± 14.41	189.00 ± 155.68	19.30 ± 3.66			
FHH-5BN		174.1 ± 23.69	434.8 ± 181.5	25.89 ± 5.95			
FHH-9BN		174.85 ± 15.81	514.80 ± 116.21	19.96 ± 4.56			
FHH-15BN		160.32 ± 17.94	232.75 ± 133.08	21.01 ± 4.55			
FHH-16BN	135.36 ± 23.04	165.26 ± 35.70	239.42 ± 233.71	7.80 ± 9.00			
FHH-18BN	126.74 ± 9.48	182.05 ± 12.83	424.91 ± 167.35	11.86 ± 7.36			
FHH-20BN	127.33 ± 12.49	148.576 ± 14.08	282.07 ± 170.22	26.38 ± 5.17			


	Low salt, MAP (mmHg)	8% salt			4% salt		
		MAP (mmHg)	UPE (mg/day)	Na+ excretion (mEq/day)	MAP (mmHg)	UPE (mg/day)	Na+ excretion (mEq/day)
SS^b^	131.32 ± 9.60	176.81 ± 20.06	215.00 ± 116.41	14.23 ± 7.78	157.81 ± 25.64	132.70 ± 95.13	13.82 ± 4.11
SS-1BN		130.5 ± 4.35	48.17 ± 14.26	15.6 ± 7.57			
SS-5BN	123.9 ± 3.85	134.0 ± 6.85	46.14 ± 23.12	20.67 ± 5.60			
SS-9BN	121.72 ± 5.53	161.20 ± 9.28	170.39 ± 75.12	18.68 ± 7.78	131.66 ± 6.44	41.47 ± 20.18	12.70 ± 5.55
SS-15BN		159.66 ± 14.91	139.22 ± 94.58	20.45 ± 5.78			
SS-16BN	125.86 ± 10.00	155.24 ± 8.70	145.28 ± 75.15	25.69 ± 7.85	136.50 ± 12.21	49.72 ± 24.18	18.33 ± 1.92
SS-18BN	123.95 ± 14.93	134.57 ± 7.13	51.57 ± 22.65	12.51 ± 2.97	137.99 ± 11.96	77.48 ± 72.13	13.00 ± 2.24
SS-20BN	122.59 ± 11.01	147.43 ± 20.57	134.48 ± 57.45	15.96 ± 6.20	138.36 ± 11.42	43.64 ± 27.67	12.74 ± 11.82


^a^FHH-1BN, official symbol FHH-Chr1^BN^/Mcwi (RGD:629520), transfer of chromosome 1 from BN to FHH genetic background.^b^SS-1BN, official symbol SS-Chr1^BN^/Mcwi (RGD: 1600490), transfer of chromosome 1 from BN to SS genetic background.Values are means ± standard deviation. All the data were downloaded from the expanded data tables in PhenoMiner.

### Congenic and mutant rats

A systemic search was made to find congenics and mutants for the seven chromosomes listed in Table 3. There are SS congenics of chromosomes1, 5, 16 and 18, among these four chromosomes, only chromosomes 1 and 5 have mutant rats. We thus focused on chromosomes 1 and 5 for the blood pressure and renal records.

### Chromosome 1 congenic rats

**Figure 3.**
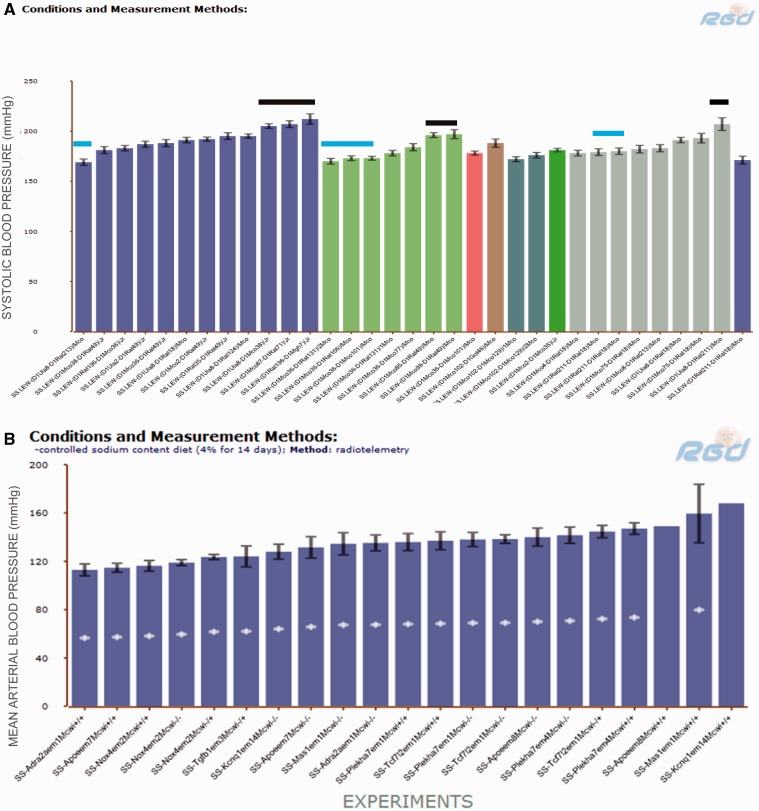
(**A**) SBP of SS.LEW congenic rats from PhenoMiner. Black horizontal bars indicate congenics that had comparable SBP to the parents and blue horizontal bars indicate congenics that had lower SBP than the parents. (**B**) MAP of SS chromosome 1 mutants from PhenoMiner. −\−, homozygous mutant; −\+, heterozygous mutant; +\+, wild-type control.

Transferring chromosome 1 from the BN rat ameliorated salt-induced hypertension in both SS and FHH backgrounds (Table 3). To narrow down blood pressure- controlling regions on chromosome 1, we looked for SS congenics containing genome elements from hypertension-resistant strains such as BN or LEW. There were diastolic blood pressure, MAP and SBP records from SS.LEW congenics, but no blood pressure records from SS.BN congenics in PhenoMiner. The cardiovascular records were retrieved by limiting strains to congenic SS.LEW strains in which the normotensive genome fragments from LEW were introgressed onto the hypertension-susceptible SS background. Thirty-three records curated from five publications were available from PhenoMiner (Figure 3A). There were six SS.LEW strains (MAP ranges from 190 to 210 mmHg, indicated by black bars) that exhibited elevated SBP comparable to the SS parents. Others, indicated by blue bars, such as SS.LEW-(D1Uia8-D1Rat213)/Mco (RGD: 4107065), SS.LEW-(D1Mco36-D1Mco101)/Mco (RGD: 2292653), SS.LEW-(D1Mco36-D1Rat106)/Mco (RGD: 2292647), SS.LEW-(D1Mco36-D1Rat131)/1Mco (RGD: 2292649) and SS.LEW-(D1Rat211-D1Rat18)/Mco (RGD: 731190) exhibited significantly lower SBP compared with SS parents(15–17). The genomic regions regulating salt-induced hypertension were mapped to D1Rat211–D1Rat18 (17) and D1Mco36–D1Mco101 (16). Users can access all the cardiovascular-related records such as MAP, heart rate or pulse pressure from these two publications on RGD reference report pages (http://www.rgd.mcw.edu/rgdweb/report/reference/main.html? id =2291850 and http://www.rgd.mcw.edu/rgdweb/report/reference/main.html?id=728381). Sequence variations among the sequenced strains in these regions can be analysed using the Variant Visualizer tool (http://www.rgd.mcw.edu/rgdweb/front/select.html) at the RGD website.

### Chromosome 1 mutant rats

**Figure 4.**
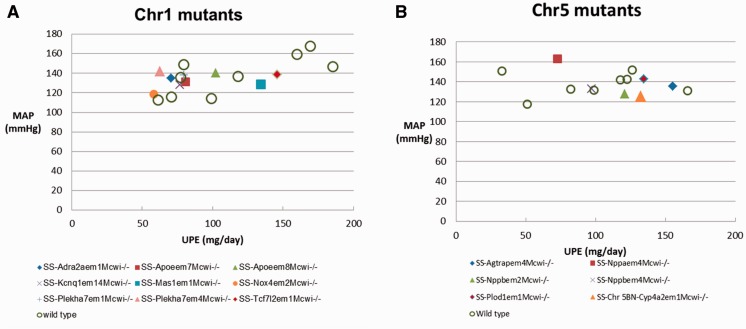
Scatterplots of daily UPE and MAP in SS mutants and wild-type controls on a 4% salt diet. Each data point is the average for a rat strain. Details are downloadable from the expanded data tables in PhenoMiner.

The blood pressures records from mutant rats allow us to come down to the gene level in the study of blood pressure regulation. The SS chromosome 1 mutant rat strains were retrieved from PhenoMiner by selecting ‘chromosome 1 mutant’ under the ‘chromosome altered’ folder on the ‘Rat Strains Selection’ page. There were 84 MAP records from SS mutants, including wild-type controls, homozygous mutants and heterozygous mutants in PhenoMiner. Figure 3B shows the MAPs in the mutants and wild-type control rats after a high salt (4% NaCl) diet for 14 days. The wild-type controls (+/+) were intermixed among mutants with MAPs ranging from 113 to 160 mmHg, with an average of 138 mmHg. To further compare the salt-induced phenotypes of homozygous mutants and controls, UPE records of these rats were retrieved and plotted against MAP in Figure 4A. Loss of NADPH oxidase 4 (Nox4) in the SS-*Nox4^em^^2^^Mcwi-/^^-^* mutants (RGD: 4139868) resulted in lower MAP and UPE compared with most of the controls and mutants, suggesting its role in salt-induced hypertension and renal damage in SS rats. Nox4 is one of the major producers of reactive oxygen species and has been associated with renal injuries in salt-induced hypertensive rats (18) and in kidney diseases in mice (19). Knocking out Nox4 in SS under a high salt diet might compromise the production of harmful reactive oxygen species and thus ameliorate the salt-induced disease phenotypes in the mutant. The transcription factor 7-like 2 (Tcf7l2) mutants (SS-*Tcf7l2^em^^1^^Mcwi-/-^*) (RGD: 5688074) and the MAS1 proto-oncogene (Mas1) mutants (SS-*Mas1^em^^1^^Mcwi-/-^*) (RGD:5688028) had the highest combined MAP and UPE among mutants, indicating those genes are protective against hypertension and renal injury.

### Chromosome 5 congenic rats

There are data from SS.LEW chromosome 5 congenic rats curated from two publications (20,21) in PhenoMiner (data not shown). From these congenic studies, the blood pressure-regulating regions on chromosome 5 were mapped to the central and telomeric regions (21–23). The Cyp4a gene family located in the central region of LEW rat chromosome 5 was identified as a blood pressure modulator (21). The Agtrap-Plod1locus located in the telomeric region, which has been implicated in hypertension and renal diseases in multiple human studies (24), was targeted for a knockout study in the PhysGen (http://www.rgd.mcw.edu/wg/physgenknockouts).

### Chromosome 5 mutant rats

There are 68 MAP records of SS chromosome 5 mutant rats, including wild-type controls, homozygous mutants and heterozygous mutants in PhenoMiner. The UPE and MAP in the six homozygous mutant rats and eight controls were plotted and compared in Figure 4B. In a comparison among the mutants, the natriuretic peptide A (Nppa) mutant (SS-*Nppa^em^^4^^Mcwi-/-^*) (RGD: 5686725) had the highest MAP, but lowest UPE, and the angiotensin II receptor-associated protein (Agtrap) mutant (SS-*Agtrap^em^^4^^Mcwi-/^*) (RGD: 5686304) had a medium MAP, but the highest UPE. Detailed analysis of the cardiovascular and renal phenotypes of these mutants and their relationship to human diseases was published by Flister *et al*. (24).

## Discussion

The FHH and SS rats developed hypertension after high salt diets, whereas BN rats did not. To find the genome elements regulating blood pressure, we used combinations of ontology terms to mine quantitative phenotype data from PhenoMiner. Data from chromosome substitution studies have demonstrated that several chromosomes play roles in the development of hypertension and renal disease in FHH (4) and SS (25) (Table 3). Subsequently from the available data, congenics and mutants of chromosomes 1 and 5 were further examined. There is good corroboration between the data retrieved from these two types of studies. The Mas 1 gene resides within the blood pressure-regulating region (D1Rat211–D1Rat18) identified in congenic studies (Figure 3A). Its role in hypertension and renal diseases has been confirmed by the exacerbated disease phenotype in the mutant strain (Figure 4A). On chromosome 5, Nppa and Agtrap are among the genes residing in the blood pressure-regulating regions, and their knockout mutants exhibit interesting phenotypes in high salt diets. Further examination of other curated cardiovascular and renal records of these mutant strains in PhenoMiner may reveal more information about how these genes modulate hypertension and renal disease in hypertensive rats.

Hypertension is an important health issue in modern society. To study the underlying mechanism of hypertension, rats have been used extensively as a disease model, especially salt-induced hypertension (5). Genetically modified rats (consomic, congenic and mutant) have been generated to map out the genomic elements regulating blood pressure. The quantitative data from these genome manipulation studies are not only useful in interpreting the original experiments but also valuable to researchers employing similar approaches. To systematically organize these data and present them in a useful way to biomedical researchers, RGD has launched PhenoMiner, a quantitative phenotype data curation project (8). The data in PhenoMiner are organized by four ontologies: RS (9), CMO, MMO and XCO (10,11). This ontological organization of phenotypic data allows users to retrieve data of interest in an efficient manner. PhenoMiner uses the RS ontology (9) to identify individual strains used in studies. Following the RS Ontology, the kinship among the rats used in different studies can be mapped out. To look for strains carrying blood pressure-regulating regions on chromosomes, users can use the ‘Rat Strain Selection Page’ in PhenoMiner to search for congenic strains or drill down to the gene level by selecting appropriate mutant rat strains. At RGD, each rat strain report page also provides links to curated data under the ‘Phenotype Values via PhenoMiner’ tab. Each link brings up the PhenoMiner tool with all its functionality such as chart display and data download. Users can download the experimental details associated with each record such as animal age, sex, sample size and the references from which the data were curated. The reference link enables users to find related data from the curated publication. The curated data link is provided under the ‘Experimental Data Annotations’ tab on the reference report page (e.g. http://www.rgd.mcw.edu/rgdweb/report/reference/main. html?id=1578407). PhenoMiner data can also be accessed from ontology terms used in data curation. For example, the ‘urine sodium excretion rate’ ontology report page (http://www.rgd.mcw.edu/rgdweb/ontology/annot.html?acc_id=CMO:0000760) lists related phenotype data for all associated RS, CMO, XCO and MMO terms in PhenoMiner.

PhysGen and NBRP are the two major high-throughput data contributors, and their data are complementary to one another. There are more than 30 000 PhysGen and PhysGen Knockout records imported into PhenoMiner. These are quantitative phenotypes measured from a variety of rat strains, including wild-type parents, consomics, congenics and mutants, with a focus on hypertension and renal diseases. On the other hand, NBRP collects phenotype data under control conditions and as such provides basal values for physiological measurements. The manual annotations in PhenoMiner were curated from more than 290 papers and more than 40% of them report cardiovascular research. RGD intends to expand its literature coverage to capture the complete spectrum of rat physiology research. Future curation targets include development, behaviour, pharmacogenomics and chemical interactions.

## Summary

The goal of the PhenoMiner project is to provide the research community with organized and retrievable quantitative phenotype data curated from different rat research laboratories. The data currently comprise results from NBRP, the PhysGen projects and the biomedical literature manually curated by RGD. Future content will include expanding manual curation coverage, results from the MCW Gene Editing Rat Resource Centre (http://www.rgd.mcw.edu/wg/gerrc), an ongoing community-engaged rat genome-modifying project and data directly submitted from laboratories involved in rat research. In the PhenoMiner user-interface, all records are searchable by controlled vocabularies and can be downloaded for further reference. Using salt-induced hypertension as an example, we demonstrated how the data in Phenominer can be searched and analysed using the functionality available in the tool.

## Funding

This work was supported by the National Heart, Lung and Blood Institute on behalf of the National Institutes of Health (HL64541). Funding for open access charge: The National Heart, Lung and Blood Institute on behalf of the National Institutes of Health (HL64541).

*Conflict of interest.* None declared.
